# Nursing practices in Primary Health Care in rural areas: a scoping review

**DOI:** 10.1590/0034-7167-2025-0592

**Published:** 2026-10-16

**Authors:** Jôse Ribas Galvão, Márcia Cristina Rodrigues Fausto, Lucas Manoel da Silva Cabral, Patty Fidelis de Almeida

**Affiliations:** I Universidade Federal da Bahia. Vitória da Conquista, Bahia, Brazil; II Fiocruz, Escola Nacional de Saúde Pública Sergio Arouca. Rio de Janeiro, Rio de Janeiro, Brazil; III Fiocruz, Instituto Leônidas & Maria Deane. Manaus,Amazonas, Brazil; IV Universidade Federal Fluminense, Instituto de Saúde Coletiva.Niterói, Rio de Janeiro, Brazil

**Keywords:** Nursing, Nursing, Private Duty, Advanced Practice Nursing, Primary Health Care, Rural Health Services., Enfermería, Práctica Privada de Enfermería, Enfermería de Práctica Avanzada, Atención Primaria de Salud, Servicios de Salud Rural.

## Abstract

**Objectives:**

to identify and analyze the scope of practices and strategies developed by nurses in Primary Health Care in rural areas.

**Methods:**

a scoping review was conducted in the MEDLINE, Embase, and VHL databases, which included articles published in the last ten years.

**Results:**

forty-eight articles were included, mostly from Australia and predominantly qualitative in approach. The main practices identified were: case management; health promotion and disease prevention practices; care for people with chronic diseases; practices mediated by Information and Communication Technologies; home visits; service management; medication prescription; practice transfer between professionals; emergency care; and palliative care.

**Final Considerations:**

nurses in rural Primary Health Care develop a wide range of practices, essential for expanding and improving access to health. However, they face challenges related to the lack of continuing education, poor healthcare service infrastructure, and a significant workload.

## RESUMEN

**Objetivos:**

identificar y analizar el alcance de las prácticas y estrategias desarrolladas por el personal de enfermería en Atención Primaria de Salud en zonas rurales.

**Métodos:**

revisión del alcance en las bases de datos MEDLINE, Embase y BVS, incluyendo artículos publicados en los últimos diez años.

**Resultados:**

se incluyeron cuarenta y ocho artículos, en su mayoría procedentes de Australia y con un enfoque predominantemente cualitativo. Las principales prácticas identificadas fueron: gestión de casos; prácticas de promoción de la salud y prevención de enfermedades; atención a personas con enfermedades crónicas; prácticas mediadas por las Tecnologías de la Información y la Comunicación; visitas domiciliarias; gestión de servicios; prescripción de medicamentos; transferencia de prácticas entre profesionales; atención de urgencias; y cuidados paliativos.

**Consideraciones Finales:**

las enfermeras de Atención Primaria en zonas rurales desarrollan una amplia gama de prácticas, esenciales para ampliar y mejorar el acceso a la salud. Sin embargo, se enfrentan a desafíos relacionados con la falta de formación continua, la precariedad de la infraestructura de los servicios de salud y una carga de trabajo considerable.

## INTRODUCTION

The structure of the Brazilian health system, through its principles and guidelines, constitutes fertile ground for the adoption of new professional practices, especially among nurses^(1)^. The complex health needs faced in Primary Health Care (PHC) rely decisively on nursing’s expanded role, which is responsible for assuming strategic functions in longitudinal follow-up, case management, and health promotion, contributing directly to addressing the population’s health demands^(2)^. In Brazil, PHC expansion, especially through the Family Health Strategy (FHS) since 1994, has boosted an important field of work for nurses, contributing to their active participation in the construction and consolidation of the Unified Health System, through their work in the political, social and health spheres, disseminated throughout the national territory^(3)^.

Internationally, there is a strong movement to expand nursing practices in response to the challenges of attracting and retaining the workforce and the aging population coupled with the rise in chronic diseases, which necessitates the involvement of more professionals who can provide better healthcare services. Expanding the scope of nursing practices yields results, especially in PHC, such as increased access, improved quality of care, and reduced costs, contributing to improved overall health and user satisfaction^(4)^.

In Brazilian rural settings, PHC often constitutes the main healthcare service available^(5)^. However, communities and teams face numerous challenges in developing expanded and effective practices, stemming from problems related to the availability and sufficiency of resources, working conditions, continuing education, a culture of practice assessment, and healthcare professional attraction and retention^(5,6)^. In these settings, nursing professionals stand out by developing care strategies and actions aimed at preventing discontinuity of care, especially in cases of physician vacancies, a fairly common situation^(5)^.

Similarly, studies highlight nursing’s leading role in PHC teams, as it constitutes an important pillar of the FHS and operates through different roles, whether in management, training, or health education^(7,8)^. Furthermore, this professional’s role in mediating between the team and users is noteworthy, as is their commitment to providing support and creating care strategies, even in unfavorable contexts^(7)^.

In the country, some policies to increase the healthcare workforce, especially in remote and rural areas, such as the *Mais Médicos* Program in Brazil, have been underway since 2013^(9)^. However, increasing the number of physicians alone has not been enough to expand access to and improve PHC’s effectiveness, making it urgent to discuss expanding other professionals’ practices, such as nurses^(10)^.

The topic of “Advanced Nursing Practice” is a frequent subject of debate in Brazil, especially since the Pan American Health Organization promoted it in conjunction with the Federal Nursing Council (COFEN - *Conselho Federal de Enfermagem*) and the Brazilian Nursing Association. The goal was to discuss expanding the scope of practices. In line with this movement, in 2018, COFEN conducted extensive national research to create a diagnosis of nursing practices in PHC, identifying those considered advanced or good practices. The results indicate that a large portion of the practices labeled as “advanced” were implemented nationally, with nurses responsible for a wide range of functions in PHC^(10)^.

Therefore, several studies have suggested further investigations into the scope of nurses’ practices in PHC^(3,4,11-13)^. The scope of practice is defined as a set of functions, activities, and responsibilities performed by healthcare professionals, which depend on the degree of autonomy, regulation, organizational context, and interprofessional relationships^(14)^.

Considering the importance of nurses in expanding access to healthcare in PHC, it is crucial to investigate their practices. It is hoped that this study can contribute to strengthening health policies, improving professional training, and fostering strategies that enhance and value nursing’s role in rural settings.

## OBJECTIVES

To identify and analyze the scope of practices and strategies developed by nursing professionals in PHC in rural areas. Additionally, to map the challenges encountered for the full development of these actions.

## METHODS

### Study design

A scoping review was developed to systematize knowledge available in scientific literature in a rigorous, transparent, and reliable manner, encompassing the JBI methodological recommendations^(15)^. The aim was to map and explore national and international evidence on health practices and strategies implemented by nurses in rural PHC as well as to identify knowledge gaps.

### Eligibility criteria

The review was guided by the following question: what is the scope of health practices and strategies developed by nurses working in PHC in rural areas? The acronym PCC was used, corresponding to: Participants (P) - nurses; Concept (C) - health practices; and Context (C) - PHC in rural areas.

National and international studies were included. It is worth noting that, in some countries, certified nurse-midwives are nurses who frequently work in maternal and childcare. Therefore, studies involving these professionals were included. Furthermore, studies involving advanced nurse practitioners (ANPs) and nurse practitioners were selected. Another observation refers to the diverse configuration of PHC, depending on the social protection model and health system of each country. Thus, studies where nurses’ work context was community-based or territorial-based services were included.

The review included full texts of articles published in the last ten years, without language restrictions. Research considering nurses’ role at other levels of care, as well as dissertations, theses, and other investigations, were excluded.

### Search strategy

Descriptors and their respective synonyms used are registered in the Medical Subject Headings (MeSH) and the Health Sciences Descriptors (DeCS). The model adopted for the search strategy corresponded to that formulated for the PubMed database: (“Nursing”[Mesh] OR Nursings OR “Nurses”[Mesh] OR Nurse OR (Nursing Personnel) OR (Personnel, Nursing) OR (Registered Nurses) OR (Nurse, Registered) OR (Nurses, Registered) OR (Registered Nurse) OR “Family Nurse Practitioners”[Mesh] OR (Family Nurse Practitioner) OR (Nurse Practitioner, Family) OR (Nurse Practitioners, Family)) AND (“Nursing, Private Duty”[Mesh] OR (Private Duty Nursing) OR (Nursing, Private Practice) OR (Private Practice Nursing) OR “Nurse’s Role”[Mesh] OR (Nurse* Role) OR (Nurse Scope, Practice) OR (Nurses’ Role*) OR (Nurses Roles) OR (Nurse’s Roles) OR (Nurse’s Scope of Practice) OR (Nurses Scope, Practice) OR (Practice Nurse’s Scope*) OR (Role*, Nurses) OR (Role*, Nurses’) OR (Role*, Nurse’s) OR “Advanced Practice Nursing”[Mesh] OR (Nursing, Advanced Practice) OR (Practice Nursing, Advanced))) AND (“Primary Health Care”[Mesh] OR (Care, Primary Health) OR (Health Care, Primary) OR (Primary Care) OR (Care, Primary) OR (Primary Healthcare) OR (Healthcare, Primary))) AND (“Rural Health Services”[Mesh] OR (Center*, Rural Health) OR (Health Center*, Rural) OR (Health Service*, Rural) OR (Rural Health Center*) OR (Rural Health Service) OR (Service*, Rural Health). The corresponding translation of the descriptors was used in each database.

The information sources used for the review were the Medical Literature Analysis and Retrieval System Online (MEDLINE), the Excerpta Medica database (Embase), and the Virtual Health Library (VHL).

### Data collection

After processing the search strategy, in August 2024, the resulting studies were imported into Rayyan Intelligent Systematic Review. This web platform assists researchers in organizing material and screening studies using a semi-automated process^(16)^. In the tool, duplicate articles were excluded (Figure 1).

**Figure 1:**
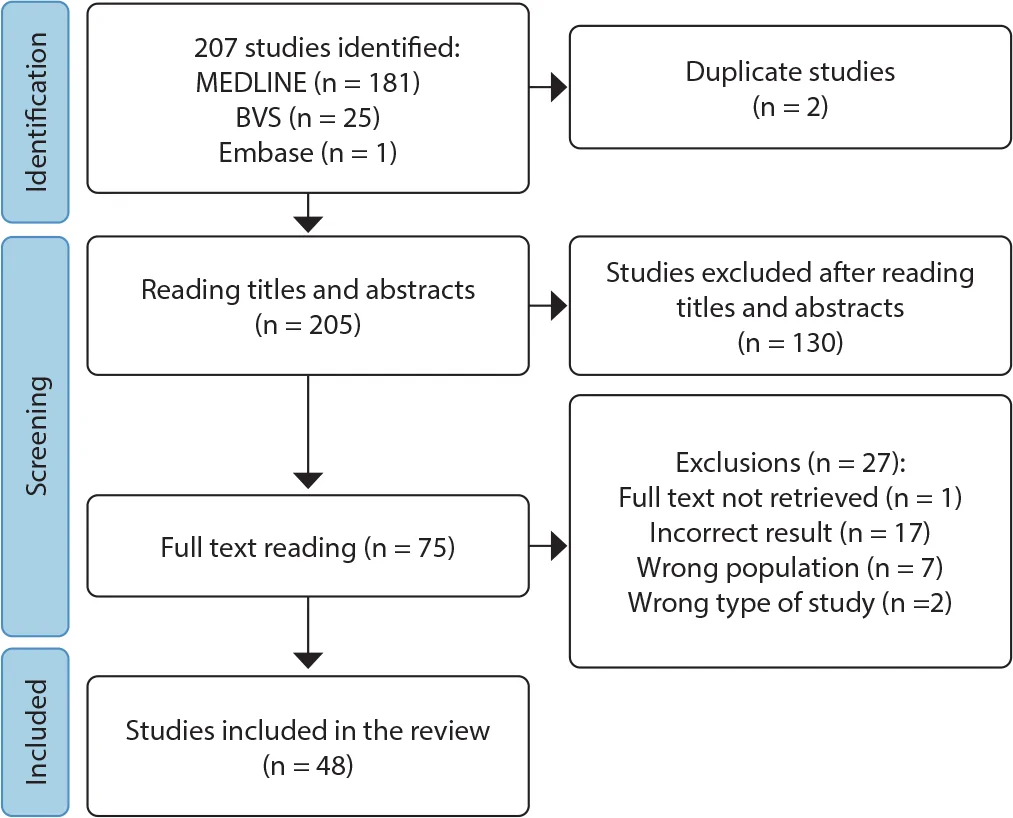
Flowchart of the study selection stages for scoping review, Vitória da Conquista, Bahia, Brazil, 2024

Subsequently, the studies were reviewed by two reviewers independently reading titles and abstracts. Discrepancies were analyzed by a third reviewer. Then, the studies were read in full by the reviewers and selected according to the inclusion criteria.

### Data analysis

The selected productions were imported into a Microsoft Excel^®^ spreadsheet, in which information was systematized regarding the study title, author(s), year of publication, country, objective, methodological design, target population of the intervention, rural PHC nurses’ health practices, results achieved, and initiatives that contributed to overcoming inequalities in access and guiding public policies, challenges, and recommendations.

Material processing was guided by content analysis^(17)^, comprising the following stages: material pre-analysis; classification into categories; and vertical and horizontal content analysis.

## RESULTS

A total of 207 studies were identified, with two removed due to duplication. Through reading titles and abstracts, 75 were selected for full-text reading. At this stage, one work was excluded for not having a complete text, despite the application of a filter, and 26 for not meeting the objectives of the review, as they did not discuss nursing practices, involved nurses’ performance at another level of care, and were in the format of a dissertation, thesis, or document (Figure 1).

Most studies originated from Australia (18.7%), the United States (16.7%), Canada (10.4%), and Brazil (8.3%). The highest number of studies was observed from 2019 onwards. The most frequently used methodological approach was qualitative (54.2%), followed by quantitative (25%) and mixed methods (20.8%) (Chart 1).

**Chart 1:** Description of the studies included in the review - author, year, country of study, and study objective and design, Vitória da Conquista, Bahia, Brazil, 2025

AUTHOR/YEAR/COUNTRY	STUDY OBJECTIVE	STUDY DESIGN
Lima *et al.*/2023/Brazil	Identify the specificities and strategies of the organization of Primary Health Care in remote rural municipalities of western Pará, considering the unique characteristics of the Amazonian context.	Qualitative, multiple case study
Paula *et al.*/2022/Brazil	Identify in the literature aspects related to the sexual and reproductive health of women living in rural areas.	Qualitative, integrative review
Rojas *et al.*/2021/Peru	Describe nursing professionals’ experiences during home visits as a Primary Health Care strategy.	Qualitative, descriptive
Oliveira *et al.*/2018/Brazil	Analyze the daily work routine of nurses in the Family Health Strategy who work in rural areas.	Qualitative, descriptive-exploratory
Borges *et al.*/2016/Brazil	Analyze the conceptions of professionals in the rural Family Health Strategy regarding the management of work in health.	Qualitative
Mallow *et al.*/2018/United States	Present the initial effectiveness of mI SMART for biophysical outcomes in people with multiple chronic conditions living in rural areas.	Quantitative, prospective pre-/post-intervention
Davidson and Reidlinger/2022/ Australia	Provide an overview of the development of nursing practices that can be translated to strengthen nutritionists’ role in Primary Health Care.	Qualitative, scoping review
Johansen and Ervik/2022/ Norway	Explore how rural healthcare professionals experience local and regional collaboration for patients in need of palliative care.	Qualitative
McCullough *et al.*/2022/ Australia	Discuss why the generalist scope of practice of nurses in remote areas should be formally recognized as an advanced and specialized nursing practice area.	Qualitative, Grounded Theory methodology
Aytona *et al.*/2022/Philippines	Estimate the need for a health workforce based on actual workloads of healthcare professionals.	Mixed, cross-sectional
Van Heerden and Jenkins/2022/South Africa	Explore community health workers’ role in providing palliative care in a rural subdistrict in South Africa.	Qualitative, descriptive-exploratory
Muench *et al.*/2022/United States	Examine rural/urban prescribing of psychiatric medications for older adults with severe mental illness by primary care providers and behavioral health specialists.	Quantitative, descriptive-retrospective
Martin-Misener/2020/Canada	Examine the context of practice and responsibilities of nurses providing essential primary care in rural and remote Canada.	Quantitative, cross-sectional
Yonge *et al.*/2019/Canada	Explore how rural nurses and students define and deal with the risks and burdens of road travel in rural areas.	Qualitative, collaborative
Zakumumpa/2020/Uganda	Describe how nurses’ role has expanded in Ugandan health facilities during the era of antiretroviral therapy expansion.	Mixed, cross-sectional, and multiple case studies
Kobe *et al.*/2022/United States	Examine the implementation of Advanced Comprehensive Diabetes Care, a comprehensive, evidence-based telehealth intervention for type 2 diabetes.	Mixed, intervention implementation study
Garg *et al.*/2022/India	Assess nurses’ competence in the Indian state of Chhattisgarh.	Quantitative
Burrows, Calleja and Cooke/2019/Australia	Determine the support needs of nurses providing emergency care in rural settings.	Qualitative, scoping review
Breton *et al.*/2023/Canada	Identify the advanced access profiles of Primary Health Care providers.	Quantitative, cross-sectional
Neville *et al.*/2020/Australia	Explore how older adults express themselves regarding access to rural community healthcare services.	Qualitative, narrative gerontology
Nelson-Brantley *et al.*/2021/United States	Describe common strategies and specific practice barriers, adaptations, and determinants of cancer screening implementation in rural Primary Health Care following adherence to a responsible healthcare organization strategy.	Qualitative, multiple case study
Haykin *et al.*/2020/Ghana	Explore the perceptions of non-medical healthcare professionals regarding their ability to manage cardiovascular diseases and the barriers to implementing the World Health Organization’s HEARTS intervention package in Community-based Health Planning and Services initiative facilities.	Qualitative
Norman *et al.*/2023/New Zealand	Explore rural nurses’ experiences in the face of barriers to providing healthcare for weight management.	Qualitative, case study
Strachan *et al.*/2022/Scotland	Assess the implementation of advanced nurses’ role in Primary Health Care and establish what works, for whom, why, and in what context.	Qualitative, multiple case study
Pace *et al.*/2018/Rwanda	Assess the impact of training on breast cancer detection on the knowledge and skills of nurses in Primary Health Care and rural community health workers.	Quantitative, evaluative
Lee *et al.*/2016/United States	Assess a centralized electronic consultation program (E-consult) on the accessibility of rural patients and the impact on osteoporosis assessment and treatment.	Quantitative, evaluative
Osborn *et al.*/2022/Australia	Assess the accessibility, availability, and utilization of community healthcare services for Aboriginal peoples and describe factors that contribute to providing effective healthcare services from providers’ perspective.	Mixed, case study
Coleman-Minahan *et al.*/2020/United States	Examine the interest of advanced practice clinicians in training to provide medication and aspiration abortion in Colorado.	Quantitative
Kwak, Hwang and Lee/2021/Korea	Explore the effects of a chronic disease management program using the physician-nurse telemedicine model in Primary Health Care and the effects on medication adherence and health-related quality of life in patients with chronic diseases from remote rural areas.	Quantitative, quasi-experimental preand post-test model with non-equivalent control group
Amin *et al.*/2020/India	Identify the needs to ensure the availability and effectiveness of Primary Health Care nurses in providing healthcare in rural areas.	Mixed, rural path approach
Johns, McAuliffe and Dorsett/2019/Australia	Investigate the provision of psychosocial care for users in palliative care in rural Australian communities, as well as identify barriers and facilitators of referrals to social services.	Qualitative
Xue *et al.*/2017/United States	Document temporal trends in alternative models of primary care for older adults and examine the role of these models in the care of rural older adults with multiple chronic conditions.	Quantitative, cross-sectional
Foster at al./2018/Zambia	Assess the results of a training program in leadership and management practices for rural nurses.	Mixed, evaluative
McCullough *et al.*/2020/Australia	Describe and explain, from nurses’ perspective, the actions and interactions involved in the provision of Primary Health Care in remote communities.	Qualitative
Diema *et al.*/2021/Ghana	Describe home visiting practices in a rural district in the Volta region of Ghana.	Mixed, descriptive cross-sectional
Suk, Oh and Im/2018/South Korea	Examine the levels of cultural competence, empowerment, and empathy in visiting nurses working with rural multicultural families in South Korea, as well as verify the factors that affect cultural competence.	Quantitative, descriptive cross-sectional
Utz *et al.*/2020/Morocco	Assess the acceptability of the screening and management approach for gestational diabetes mellitus by healthcare providers.	Qualitative, evaluative
Hjertstrøm, Obstfelder and Norbye/2018/Norway	Analyze the efforts of Norwegian nursing leaders in developing a rural municipal health emergency service.	Qualitative, ethnographic approach
Lovero *et al.*/2019/South Africa	Assess the progress and challenges in implementing the South African National Mental Health Policy and Strategic Plan 2013-2020.	Mixed, evaluative
Hungerford *et al.*/2016/Australia	Explain the main components of the nursing practice model developed to meet the health needs of a remote and tourist community (without a resident general practitioner).	Qualitative, case study
Singh *et al.*/2019/India	Assess the perspectives of rural providers on the management and referral of pregnant women with high obstetric risk or complications.	Quantitative, cross-sectional
Jin *et al.*/2019/China	Explore the association between expanded roles and job satisfaction of rural Primary Health Care providers in China.	Mixed, cross-sectional
Roots and MacDonald/2014/Canada	Identify outcomes associated with the nurses’ role in collaborative practice in Primary Health Care.	Qualitative, multiple case studies
Reid, Ervin and Kelly/2019/Australia	Describe the first year of implementation of a physician-in-high school program in a rural setting in Victoria, Australia.	Qualitative, descriptive with a pragmatic approach
Iliffe *et al.*/2014/United Kingdom	Adapt and test a North American case management model based on primary care for people with dementia.	Mixed, multiple case studies
Mothiba and Tladi/2016/ South Africa	Determine the challenges faced by nurses when implementing the Expanded Immunization Program in rural clinics in the Capricorn District, Limpopo Province, South Africa.	Qualitative, descriptive exploratory
Pesut *et al.*/2017/Canada	Test a rural palliative care service led by nurses.	Mixed, single-center observational
Biordi *et al.*/2015/United States	Present a model for the provision of oral healthcare services in two locations of the Special Program for Supplementary Nutrition for Women, Infants and Children.	Quantitative

Notable studies included intervention assessments targeting individuals with multiple chronic conditions, refractory type 2 diabetes mellitus, women undergoing screening for breast cancer and other breast diseases, cardiovascular diseases, dementia, older adults with advanced chronic disease, and children under 5 years of age. Investigations that did not involve intervention assessment mostly focused on the care of people with chronic diseases or older adults. Research on policy and program implementation was also found.

Chart 2 presents the health practices and strategies developed by rural PHC nurses, identified and categorized through thematic analysis, followed by the total number of studies that addressed them.

**Chart 2:** Health practices and strategies developed by nurses working in Primary Health Care in rural areas, Vitória da Conquista, Bahia, Brazil, 2025

PRACTICES/STRATEGIES	TOTAL NUMBER OF STUDIES
Care coordination/case management	17
Health promotion, disease prevention, and intersectoral practices	15
Care for people with chronic diseases	14
Information and Communication Technology-mediated practices	12
Home visits	12
Women’s healthcare	12
Medication prescription and treatment	11
Service management	11
Collaborative practices with other professionals	8
Transfer of practices between professionals	6
Emergency care	5
Palliative care	4
Assistance to people living with HIV	3
Preceptorship	1

Despite the widespread role of nurses in rural areas, studies have shown several challenges to their full development, as illustrated in Figure 2.

**Figure 2:**
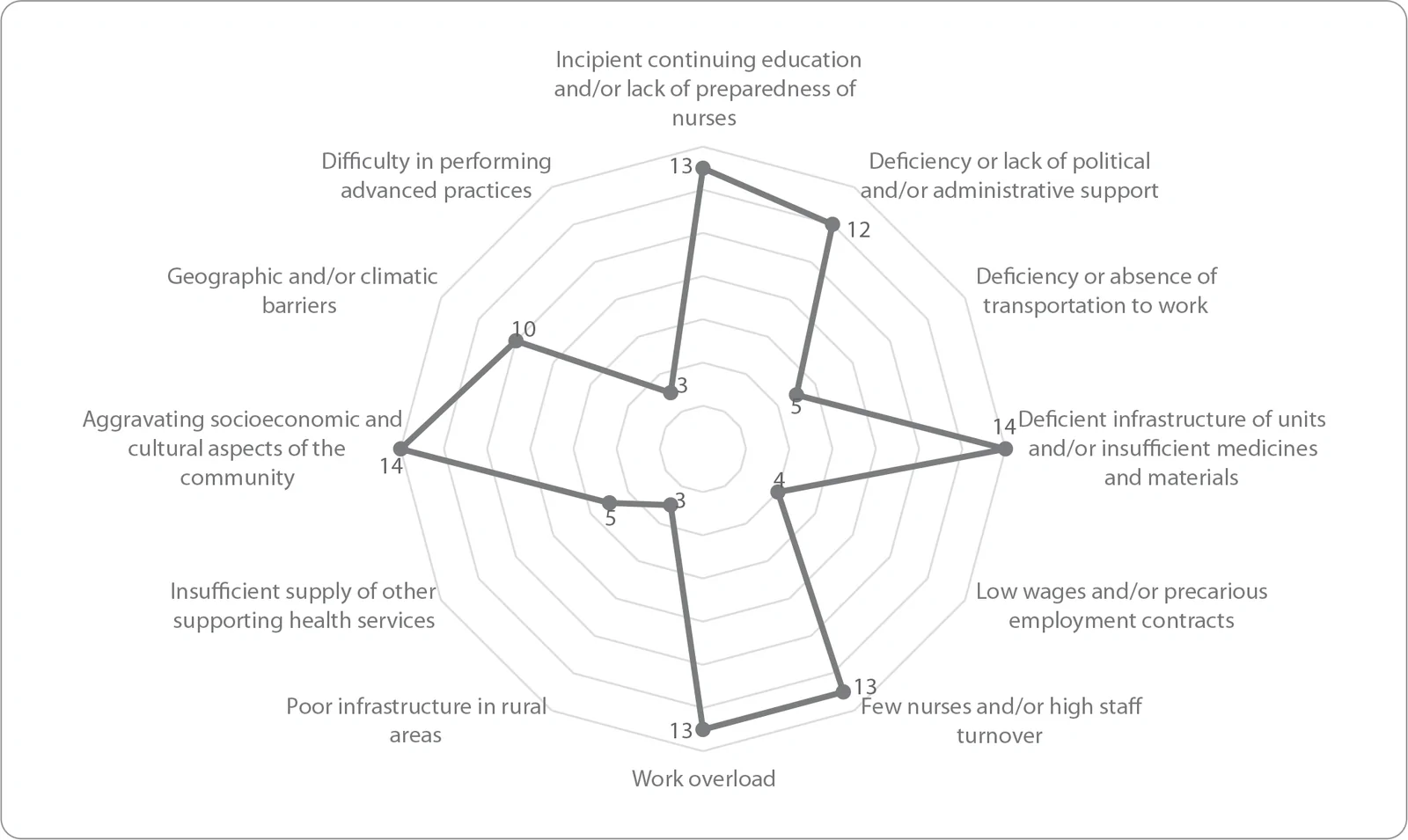
Challenges faced by nurses working in Primary Health Care in rural areas, Vitória da Conquista, Bahia, Brazil, 2025

## DISCUSSION

In general, through thematic analysis of studies, it is possible to identify a wide scope of practices and strategies undertaken by nurses. The rural context, coupled with the lack or turnover of medical professionals, presents PHC nurses with the need to develop a mosaic of practices, often overlapping, which requires knowledge and skills quite different from those existing in other environments, justifying the definition of the “remote area nurse” as a specialized function^(18)^.

Transfer of practices (task shifting or skill mix) involves the assumption of functions by other professionals, resulting in an expansion of the scope and increased access to services by users. This process was common^(18-21)^ and considered strategic to improve access to PHC for rural populations. This practice did occur even though it was not formalized in the country; in other words, it involved practices not covered by the legal scope of nursing, driven by the specific needs of rural areas and the absence of other professionals^(19-21)^. One example was the management of care for people with HIV, including prescribing antiretroviral therapy (ART) and performing circumcision surgery, in Uganda, traditionally assigned to physicians^(19)^. In experiments conducted in Australia, nurses worked alone in an emergency department, carrying out patient recovery, screening, stabilization, and continuation of treatment until discharge^(18)^. In Canada, they independently requested and interpreted tests, made diagnoses, prescribed medications, and made referrals^(21)^.

Nurses commonly performed the duties not only of physicians, but also of pharmacists, laboratory technicians, paramedics, administrators, radiologists, social workers, and physiotherapists, under conditions of low wages and excessive workload^(21)^. Studies suggest reviewing the scope of nurses’ practice regarding task transfer, describing their roles and policies, to formalize these practices and ensure patient safety and quality of care through appropriate educational processes. Furthermore, increasing the number of nurses is recommended to reduce overlapping roles^(19-21)^.

Initiatives for collaborative practices by nurses with other professionals^(22-27)^ were a finding and, when interinstitutional, operated by videoconferences and regular meetings. Strategies for coordination between PHC nurses and home care nurses, nursing home teams, and general practitioners to establish a rural emergency service with care coordination, in a Norwegian experience, facilitated user access and contributed to comprehensive care^(25)^.

Collaborative practices between nurses and other professionals in PHC have involved: community health workers (CHWs) and multidisciplinary team professionals in home visits, case studies, and educational groups in Brazil^(27)^; medication manager in telehealth interventions for patients with diabetes in the United States ^(22)^; physicians providing healthcare services in schools in Australia^(23)^; and several members of the PHC team involved in planning, assessment, monitoring of actions, and shared care in other settings^(24-26)^. In the Canadian case, collaborative practices included shared care and resulted in improved interprofessional communication and teamwork^(26)^.

Rural nurses frequently worked night shifts, often being the only healthcare provider available during that time. During their shifts, they were responsible for emergency care, monitoring patients in emergency beds until stabilization, and transporting patients^(18,25,27,28)^.

In a Norwegian initiative^(25)^, nurses, in addition to managing emergency care, operated a local emergency communication center, where patients were screened by telephone to decide whether face-to-face care or counseling was necessary, which required a set of skills and knowledge not always covered by training processes. Despite rare continuing education initiatives, an experience in Scotland^(28)^ deserves mention, in which entities responsible for the management and assistance of National Health Service health boards, in remote and rural areas, offered advanced nursing training specifically for emergency care.

The care provided by nurses to people with chronic diseases was frequent^(18,19,22,26,29,30)^, as well as older adults and palliative care. Cases of assistance to people living with HIV occurred in Uganda^(19)^, Zambia^(30)^, and Canada^(26)^, including ART prescription and counseling, medication dispensing, screening, blood collection for laboratory tests, HIV testing, treatment room work and tuberculosis case management.

In the Ugandan case, nurses led home visits, during which ART was offered with the aim of relieving pressure on HIV clinics and reducing patient transportation costs^(19)^. In the Zambia experience, the entire team, including CHWs and volunteers, was trained in counseling and testing for sexually transmitted infections, which resulted in improvements in service accessibility (e.g., an increase in the availability of HIV testing for babies exposed to HIV from 60% to 83%)^(30)^.

Palliative care encompassed actions such as developing a care plan, providing psychosocial care, conducting home visits, supervising CHWs for continuous patient monitoring, and providing educational activities for caregivers and patients^(24,31)^. In Norway, rural primary care nurses facilitated regular video conferences with specialists to discuss the clinical situation of people in palliative care, make joint decisions, and clarify doubts, which sometimes avoided the need for patients to travel to hospitals^(24)^.

A pilot study in Canada^(31)^ tested a rural palliative care service for older adults led by nurses. The nurse-led navigation incorporated into care, in addition to healthcare services, social assistance actions (mobilization of resources for transport, meals, and cleaning) and coordination with support networks. Older adults and caregivers were offered a 24-hour telephone helpline, available every day of the week, for nursing care between visits, and a diary to record relevant health issues of users, in order to share with other care providers. The nurse-led navigation sent reports to the attending physicians with the clinical progress and made referrals when necessary^(31)^.

Another intervention created and led by nurses for people with multiple chronic conditions, in the United States^(32)^, was mI SMART, a web-based mobile sensor and device framework using the Chronic Care Model. The mI SMART patient functionality combined synchronous and asynchronous education, reminders to perform self-monitoring, self-monitoring logs with automated and personal responses from physicians, medication notifications, an asynchronous messaging portal, video conferencing for routine consultations, access to laboratory results, and links to conduct surveys. Each user received self-monitoring devices (scales, glucometer, blood pressure monitor, and tablet) and an internet data package. As a result, rural users showed improvements in glucose levels, blood pressure, and weight within a short period of 12 weeks^(32)^.

Through information and communication technologies, nurses received guidance from a physician remotely to provide care to patients with chronic diseases, make referrals^(18)^, promote self-monitoring and manage medications for patients with diabetes mellitus^(22)^, conduct electronic consultations (e-consult) for older adults with fractures^(33)^ and manage cases of people with dementia, establishing contact by telephone or letter/email^(34)^. These initiatives improved engagement in self-management and patient/nurse communication, promoting greater awareness of chronic disease control and increasing access to treatment, with a reduction in the need for travel.

The practice of home visits by nurses in rural settings aimed to assess socioeconomic conditions, identify risks, plan care according to patients’ reality and resources, provide health education, verify compliance with the vaccination schedule, administer vaccinations, monitor the growth and development of children, and perform curative actions^(26,27,29,31,35-37)^. In Ghana, nurses made visits in the morning, afternoon, and evening^(36)^, emphasizing the disease prevention process. In South Korea, visiting nurses were primarily responsible for providing care to rural multicultural families^(35)^.

Nursing practices related to women’s healthcare were a recurring finding and encompassed prenatal consultations (including diagnoses and treatments), postpartum care, family planning, assisted deliveries, abortion procedures, and screening and management of cervical cancer (with mobile Pap smear testing) and breast cancer^(26,27,30,36,38-43)^.

In Morocco, a decentralized screening and treatment program for gestational diabetes (GDM) was implemented in primary care units, where nurses performed various functions such as prenatal consultations, GDM screening and management, nutritional counseling, and contact via cell phone to clarify doubts and communicate appointments with specialists. This reduced delays in screening tests and increased program adherence and satisfaction among pregnant women^(39)^.

Medical abortion was also identified, with a recommendation to expand this practice in rural areas where there are no specialized physicians and where PHC units may be difficult to access^(43)^.

Prescription and treatment practices were frequent^(19,21,25,36,40,41,44)^. A study in the United States found that psychiatric pharmacies provided more prescriptions for psychiatric medications than specialist physicians to users in rural areas^(44)^. Prescription medication was also present in Uganda, India, Korea, Ghana, Norway, South Africa, and Australia, being intended for the treatment of fever, diarrhea, tuberculosis, chronic diseases, minor pain, emergencies, ART, and the treatment of early complications in pregnant women^(19,21,25,36,40,41)^. In India, nurses prescribed medications for various illnesses and conditions, such as diarrhea with severe dehydration, pneumonia, malaria, hypertension, diabetes, candidiasis, pre-eclampsia, scabies, poisoning, and sickle cell anemia, as a way to improve access for the population^(41)^.

The findings of this review highlight the prominent role of PHC nurses in rural areas in leadership and management roles within PHC services, HIV clinics, and mobile units (dental, breast cancer screening, and osteoporosis screening). Among the activities developed within the scope of managing these units, the following stand out: human, material and pharmaceutical resource management; training provision; immunization sector supervision; promotion of communication channels for users; coordination with other healthcare services to ensure integrated care; and assessment, planning, and implementation of strategies aimed at improving quality of care provided to the community^(19,20,22,23,25-27,30,44,45)^. However, a Brazilian experiment demonstrated that centralizing the management of PHC units in the hands of nurses led to increased workload^(45)^.

Health promotion and disease prevention were present and encompassed practices such as collective educational activities, strategies to mobilize community participation in the service, identification of risks in the territory, immunization actions, and implementation of health promotion programs (oral health, nutrition, and exercise)^(20,23,26,27,29-31,36)^. Nurses’ initiatives for intersectoral actions included offering services in schools and coordinating social assistance to promote comprehensive care^(23,31,46)^.

Beyond the practices and strategies employed by nurses working in rural areas, studies have highlighted a set of challenges that sometimes limit their scope of action. The specificities of rural life demand diverse skills and knowledge that are rarely offered to professionals through training processes, whether in undergraduate studies, continuing education, or on-the-job training^(18,19,27-29,35,40,46)^. This was a key issue highlighted in the studies: the need for adequate training for professionals^(18-21,27,29,35,36,39-44,46-49)^. Despite the limited number of initiatives, it is worth highlighting the case of Australia, where university and continuing education programs offered training and support for healthcare professionals in rural areas^(48)^. In India, nurses received mentoring and support via telehealth^(50)^. In any case, professionals reported difficulties in participating due to work overload, lack of substitutes, and difficulties with commuting.

The lack of transport to perform the work in rural areas^(27,29,36)^, intensified by extensive travel in unfavorable conditions (damaged and unmarked roads, snow or extreme heat)^(21,27-29,36,42)^ and a small number of nurses for large geographically dispersed populations^(18-21,27,36,38,47)^, was a recurring challenge. In some cases, nurses traveled or transported patients using their own resources.

Poor infrastructure in PHC services, insufficient medication, materials, and equipment were observed in the studies and influenced quality of care offered^(18,19,21,27-29,36,46,47)^. The lack of infrastructure in rural areas for nurses and their families, such as housing, employment opportunities for spouses, qualified schools for children^(50)^ and adequate wages^(19,27)^, emerged as challenges for nurses working in rural settings. In an Indian experience, attempts were made to address these problems by providing housing, wages comparable to those in city hospitals, and a well-equipped unit for all nurses^(50)^.

Advanced nursing practice was limited by legislation, acceptance by physicians and other professionals, limited community understanding, lack of supervision and competency assessment, and scarce continuing education initiatives, as already mentioned^(28,44)^. In one specific case in the United States, state regulations for the practice of prescribing medication required medical supervision^(44)^.

Studies have indicated recurring reports regarding the excessive workload of rural PHC nurses^(18,20,22,34,38,39,45-47)^. These professionals performed overlapping functions, such as caregiving and management roles, and at times were the sole provider of primary care, without commensurate social and financial recognition.

Problems related to underfunding, unavailability of healthcare services to support PHC, absence or limitation of career plans, gaps in the information system, deficient coordination, and centralized management far removed from the daily operations of PHC units negatively affected the possibilities for nursing practice in rural areas^(18,28,35,46)^.

### Study limitations

The absence of the term “scope of practice” or “health practices” in DeCS/MeSH is considered a limitation of this study, and similar descriptors should be used. The existence of diverse health systems has made it difficult to define PHC services in different countries, as well as to define the nursing profession, considered here as a higher education qualification, depending on the context. In any case, the importance of the significant body of evidence presented is highlighted, considering the limited number of studies addressing nursing professionals’ role in rural contexts in Brazil, despite the size of these territories, the distance from urban centers, the sparse population, and the large inequalities in access to public goods and services, including health.

### Contributions to nursing, health, or public policy

The findings and conclusions of this study contribute to strengthening health policies, especially PHC, as well as to supporting robust practice models and, consequently, to the provision of more equitable services, recognizing the specificities of rural territories. Furthermore, it can contribute to improving nurses’ professional training and promoting strategies that qualify and value their work in the rural context, in addition to contributing to the current debate about the scope of nursing practice.

## FINAL CONSIDERATIONS

Nurses in rural and remote areas play an essential role in expanding access to PHC in the areas of care, health promotion, and disease prevention, as well as in case management and health unit management.

Although the scope of practice is directed towards PHC users, the main groups benefiting from the care provided by nursing professionals working in rural areas were older adults, people with chronic diseases, and those in palliative care, according to the reviewed studies, i.e., the most vulnerable groups. Activities included consultations, diagnoses, examinations, prescriptions, home visits, case management, health unit administration, and intersectoral actions to ensure comprehensive care.

Despite its crucial role in PHC’s viability in rural contexts, the development of health practices by nurses faces challenges that can compromise quality of care. In light of the potential not only to expand but also to support the development of nursing practices, especially in rural areas, which are essential to guarantee access to health for vulnerable populations, public policies aimed at improving the infrastructure of healthcare services, guaranteeing continuing education, professional support, and retention policies are fundamental, ensuring conditions that favor the qualified and effective performance of these professionals.

## Data Availability

The research data are available in a repository: https://data.mendeley.com/datasets/fyk4vsyrmx/1.
