# Supplementary material for: Nursing practices in Primary Health Care in rural areas: a scoping review
Source: Rev Bras Enferm. 2026 Oct 16;79:e20250592. doi: 10.1590/0034-7167-2025-0592 (PMC13679169; doi:10.1590/0034-7167-2025-0592)
Supplement: Translation

# Translation: Práticas da enfermagem na Atenção Primária à Saúde em territórios rurais: revisão de escopo

**DOI:** 10.1590/0034-7167-2025-0592pt

**Authors:** Jôse Ribas Galvão, Márcia Cristina Rodrigues Fausto, Lucas Manoel da Silva Cabral, Patty Fidelis de Almeida

**Affiliations:** I Universidade Federal da Bahia. Vitória da Conquista, Bahia, Brasil; II Fiocruz, Escola Nacional de Saúde Pública Sergio Arouca. Rio de Janeiro, Rio de Janeiro, Brasil; III Fiocruz, Instituto Leônidas & Maria Deane. Manaus,Amazonas, Brasil; IV Universidade Federal Fluminense, Instituto de Saúde Coletiva.Niterói, Rio de Janeiro, Brasil

**Keywords:** Enfermagem, Prática Privada de Enfermagem, Prática Avançada de Enfermagem, Atenção Primária à, Saúde, Serviços de Saúde Rural.

## Abstract

**Objetivos:**

identificar e analisar o escopo de práticas e estratégias desenvolvidas pelo enfermeiro na Atenção Primária à Saúde em territórios rurais.

**Métodos:**

revisão de escopo realizada nas bases MEDLINE, Embase e BVS, que incluiu artigos publicados nos últimos dez anos.

**Resultados:**

foram incluídos 48 artigos, maior parte oriundos da Austrália e predominantemente de abordagem qualitativa. As principais práticas identificadas foram: gestão de casos; práticas de promoção da saúde e prevenção de doenças; cuidado de pessoas com doenças crônicas; práticas mediadas por Tecnologias de Informação e Comunicação; visitas domiciliares; gerência de serviços; prescrição medicamentosa; transferência de práticas entre profissionais; atendimento das urgências/emergências; e cuidados paliativos.

**Considerações Finais:**

enfermeiros na Atenção Primária à Saúde rural desenvolvem amplo escopo de práticas, essenciais para ampliação e qualificação do acesso à saúde. Contudo, enfrentam desafios relacionados à ausência de educação permanente, infraestrutura precária dos serviços de saúde, além da importante sobrecarga laboral.

## INTRODUÇÃO

A conformação do sistema de saúde brasileiro, por meio de seus princípios e diretrizes, constitui um campo fértil para a assunção de novas práticas profissionais, com destaque aos enfermeiros^(1)^. As necessidades complexas de saúde enfrentadas na Atenção Primária à Saúde (APS) se apoiam de modo decisivo no papel ampliado da enfermagem, responsável por assumir funções estratégicas no acompanhamento longitudinal, na gestão de casos e na promoção da saúde, contribuindo diretamente para a resolução das demandas de saúde da população^(2)^. No Brasil, a expansão da APS, especialmente pela Estratégia Saúde da Família (ESF), a partir de 1994, impulsionou um importante campo de trabalho para o enfermeiro, contribuindo para participação ativa na construção e consolidação do Sistema Único de Saúde, por meio de sua atuação nos âmbitos político, social e sanitário, disseminada por todo o território nacional^(3)^.

No âmbito internacional, existe um forte movimento de expansão das práticas de enfermagem diante dos desafios da atração e fixação da força de trabalho e do envelhecimento da população com o aumento das doenças crônicas, o que mobiliza a atuação de mais profissionais que combinem melhores condições na prestação dos cuidados em saúde. A ampliação do escopo de práticas do enfermeiro apresenta resultados, especialmente na APS, como aumento do acesso, melhoria da qualidade da assistência prestada e redução de custos, o que colabora para a melhoria da saúde em geral e para a satisfação dos usuários^(4)^.

No cenário rural brasileiro, a APS, frequentemente, constitui-se o principal serviço de saúde disponível^(5)^. No entanto, comunidades e equipes enfrentam inúmeros desafios para o desenvolvimento de práticas ampliadas e resolutivas, decorrentes de problemas relacionados à disponibilidade e à suficiência de insumos, às condições de trabalho, à educação permanente, à cultura de avaliação das práticas e à atração e à fixação de profissionais de saúde^(5,6)^. Nesses cenários, sobressaem-se os profissionais de enfermagem ao desenvolver estratégias de cuidados e ações com o objetivo de evitar a descontinuidade do cuidado, sobretudo na vacância de médicos, situação bastante recorrente^(5)^.

Na mesma direção, estudos evidenciam o protagonismo da enfermagem nas equipes de APS, ao se constituir um pilar importante da ESF e transitar por diferentes papéis, seja na gestão, na formação ou na educação em saúde^(7,8)^. Ademais, é notória a atuação desse profissional na mediação entre a equipe e os usuários, bem como seu compromisso em acolher e criar estratégias de cuidado, mesmo em contextos desfavoráveis^(7)^.

No país, algumas políticas para aumentar a força de trabalho na saúde, especialmente em áreas remotas e rurais, tais como o Programa Mais Médicos no Brasil, estão em curso desde 2013^(9)^. Todavia, o aumento do número de médicos isoladamente não tem sido capaz de ampliar o acesso e a resolutividade da APS, sendo premente a discussão a respeito da ampliação de práticas de outros profissionais, a exemplo dos enfermeiros^(10)^.

O tema da “Prática Avançada em Enfermagem” constitui pauta frequente no debate brasileiro, especialmente a partir do fomento da Organização Pan-Americana de Saúde, em conjunto com o Conselho Federal de Enfermagem (COFEN) e a Associação Brasileira de Enfermagem, com vistas a discutir a ampliação do escopo de práticas. Articulado a esse movimento, em 2018, o COFEN promoveu uma extensa pesquisa nacional com a finalidade de construir um diagnóstico das práticas de enfermagem na APS, identificando aquelas consideradas avançadas ou boas práticas. Os resultados sinalizam que boa parte das práticas nomeadas como “avançadas” eram executadas nacionalmente, sendo o enfermeiro responsável por um amplo leque de funções na APS^(10)^.

Por conseguinte, diversos estudos têm sugerido investigações mais aprofundas acerca do escopo de práticas do enfermeiro na APS^(3,4,11-13)^. Define-se escopo de prática como um conjunto de funções, atividades e responsabilidades, desempenhadas por profissionais de saúde, que dependem do grau de autonomia, regulamentação, contexto organizacional e relações interprofissionais^(14)^.

Considerando a relevância do papel do enfermeiro para ampliação do acesso aos cuidados em saúde na APS, é premente investigar as práticas deste profissional. Espera-se que o estudo possa contribuir para fortalecer políticas de saúde, aprimorar a formação profissional e fomentar estratégias que qualifiquem e valorizem a atuação da enfermagem no contexto rural.

## OBJETIVOS

Identificar e analisar o escopo de práticas e estratégias desenvolvidas pelo profissional enfermeiro na APS em territórios rurais. Adicionalmente, mapear os desafios encontrados para o pleno desenvolvimento das ações.

## MÉTODOS

### Tipo de estudo

Foi desenvolvida revisão de escopo com vistas a sistematizar conhecimentos disponíveis na literatura científica, de forma rigorosa, transparente e confiável, compreendendo as recomendações metodológicas do JBI^(15)^. Buscou-se mapear e explorar evidências nacionais e internacionais sobre as práticas de saúde e estratégias realizadas pelos enfermeiros na APS rural, assim como de indicar lacunas de conhecimento.

### Critérios de elegibilidade

A revisão foi guiada pela seguinte pergunta: qual o escopo de práticas em saúde e estratégias desenvolvidas pelos enfermeiros que atuam na APS em territórios rurais? Seguiu-se o acrônimo PCC, correspondendo a: Participantes (P) - enfermeiros; Conceito (C) - práticas de saúde; e Contexto (C) - APS em territórios rurais.

Foram incluídos estudos nacionais e internacionais. Cabe destacar que, em alguns países, parteiras (em inglês, *certified nurse-midwives*) são enfermeiras que atuam, frequentemente, nos cuidados materno-infantil. Logo, publicações envolvendo essas profissionais foram incluídas. Ademais, as publicações que envolveram enfermeiros de prática avançada ((EPA) em inglês, *advanced nurse practitioners*) e enfermeiros praticantes (em inglês, *nurse practitioners*) foram eleitas. Outra observação refere-se à diversidade de configuração da APS, a depender do modelo de proteção social e do sistema de saúde de cada país. Assim, foram incluídos estudos cujo contexto de atuação do enfermeiro eram serviços de base comunitária ou territorial.

A revisão envolveu textos completos de artigos publicados nos últimos dez anos e sem restrições de idioma. Pesquisas que consideraram a atuação dos enfermeiros em outro nível de atenção e investigações em formato de dissertação, tese e documentos foram excluídas.

### Estratégia de busca

Os descritores e respectivos sinônimos utilizados estão registrados no *Medical Subject Headings* (MeSH) e nos Descritores em Ciências da Saúde (DeCS). O modelo adotado para a estratégia de busca correspondeu ao formulado para a base de dados PubMed: (“Nursing”[Mesh] OR Nursings OR “Nurses”[Mesh] OR Nurse OR (Nursing Personnel) OR (Personnel, Nursing) OR (Registered Nurses) OR (Nurse, Registered) OR (Nurses, Registered) OR (Registered Nurse) OR “Family Nurse Practitioners”[Mesh] OR (Family Nurse Practitioner) OR (Nurse Practitioner, Family) OR (Nurse Practitioners, Family)) AND (“Nursing, Private Duty”[Mesh] OR (Private Duty Nursing) OR (Nursing, Private Practice) OR (Private Practice Nursing) OR “Nurse’s Role”[Mesh] OR (Nurse* Role) OR (Nurse Scope, Practice) OR (Nurses’ Role*) OR (Nurses Roles) OR (Nurse’s Roles) OR (Nurse’s Scope of Practice) OR (Nurses Scope, Practice) OR (Practice Nurse’s Scope*) OR (Role*, Nurses) OR (Role*, Nurses’) OR (Role*, Nurse’s) OR “Advanced Practice Nursing”[Mesh] OR (Nursing, Advanced Practice) OR (Practice Nursing, Advanced))) AND (“Primary Health Care”[Mesh] OR (Care, Primary Health) OR (Health Care, Primary) OR (Primary Care) OR (Care, Primary) OR (Primary Healthcare) OR (Healthcare, Primary))) AND (“Rural Health Services”[Mesh] OR (Center*, Rural Health) OR (Health Center*, Rural) OR (Health Service*, Rural) OR (Rural Health Center*) OR (Rural Health Service) OR (Service*, Rural Health). Em cada base de dados, utilizou-se a tradução correspondente dos descritores.

As fontes de informação utilizadas para a revisão foram a *Medical Literature Analisys and Retrieval System Online* (MEDLINE), a *Excerpta Medica dataBASE* (Embase) e a Biblioteca Virtual em Saúde (BVS).

### Coleta de dados

Após o processamento da estratégia de busca, em agosto de 2024, os estudos provenientes foram importados para o *Rayyan Intelligent Systematic Review*, plataforma *web* que auxilia o pesquisador na organização do material e triagem dos estudos, utilizando um processo de semiautomação^(16)^. Na ferramenta, os artigos duplicados foram excluídos (Figura 1).

**Figura 1:** Fluxograma das etapas de seleção dos estudos para a revisão de escopo, Vitória da Conquista, Bahia, Brasil, 2024

Posteriormente, foi realizada a revisão dos estudos por meio da leitura de títulos e resumos por dois revisores, de forma independente. Divergências foram analisadas por uma terceira revisora. Em seguida, as publicações foram lidas na íntegra pelas revisoras e eleitas de acordo com os critérios de inclusão.

### Análise de dados

As produções selecionadas foram importadas para uma planilha do *Microsoft Excel^®^*, na qual foram sistematizadas informações acerca do título do estudo, autor(es), ano de publicação, país, objetivo, delineamento metodológico, população-alvo da intervenção, práticas de saúde do enfermeiro na APS em territórios rurais, resultados alcançados e iniciativas que contribuíram para superar desigualdades de acesso e orientar políticas públicas, desafios e recomendações.

O tratamento do material foi orientado pela análise de conteúdo^(17)^, compreendendo as seguintes etapas: pré-análise do material; classificação em categorias; e análise de conteúdo vertical e horizontal.

## RESULTADOS

Foram identificados 207 estudos, sendo dois removidos por duplicação. Por meio da leitura de títulos e resumos, 75 foram selecionados para leitura na íntegra. Nesta etapa, um trabalho foi excluído por não dispor de texto completo, apesar da aplicação do filtro, e 26 por não contemplarem os objetivos da revisão, pois não discutiam práticas de enfermeiros, envolviam a atuação dos enfermeiros em outro nível de atenção e tinham formato de dissertação, tese ou documento (Figura 1).

A maior parte das publicações era originária da Austrália (18,7%), dos Estados Unidos (16,7%), do Canadá (10,4%) e do Brasil (8,3%). O maior número de produções foi observado a partir do ano de 2019. A abordagem metodológica mais utilizada foi a qualitativa (54,2%), seguida pela quantitativa (25%) e pela quanti-qualitativa (20,8%) (Quadro 1).

**Quadro 1:** Caracterização dos estudos incluídos na revisão - autor, ano, país de realização, objetivo e delineamento do estudo, Vitória da Conquista, Bahia, Brasil, 2025

AUTOR/ANO/PAÍS	OBJETIVO DO ESTUDO	DELINEAMENTO DO ESTUDO
Lima *et al.*/2023/Brasil	Identificar especificidades e estratégias da organização da Atenção Primária à Saúde em municípios rurais remotos do oeste do Pará diante das singularidades do contexto amazônico.	Qualitativo, estudo de casos múltiplos
Paula *et al.*/2022/Brasil	Identificar na literatura aspectos relacionados à saúde sexual e reprodutiva das mulheres que vivem no contexto rural.	Qualitativo, revisão integrativa
Rojas *et al.*/2021/Peru	Descrever as vivências do profissional de enfermagem na visita domiciliar como estratégia de Atenção Primária à Saúde.	Qualitativo, descritivo
Oliveira *et al.*/2018/Brasil	Analisar o cotidiano de trabalho de enfermeiros da Estratégia Saúde da Família que atuam em áreas rurais.	Qualitativo, descritivo-exploratório
Borges *et al.*/2016/Brasil	Analisar as concepções dos profissionais da Estratégia Saúde da Família rural sobre o gerenciamento do trabalho em saúde.	Qualitativo
Mallow *et al.*/2018/Estados Unidos	Apresentar a eficácia inicial do *mI SMART* para resultados biofísicos em pessoas com múltiplas condições crônicas que vivem em áreas rurais.	Quantitativo, prospectivo pré-/pós-intervenção
Davidson e Reidlinger/2022/Austrália	Fornecer uma visão geral do desenvolvimento de prática de enfermeiros que podem ser traduzidos para fortalecer a atuação do nutricionista na Atenção Primária à Saúde.	Qualitativo, revisão de escopo
Johansen e Ervik/2022/Noruega	Explorar como os profissionais de saúde rurais vivenciam a colaboração local e regional para pacientes que necessitam de cuidados paliativos.	Qualitativo
McCullough *et al.*/2022/Austrália	Discutir porque o escopo generalista de prática dos enfermeiros de áreas remotas deve ser formalmente reconhecido como uma área de prática de enfermagem avançada e especializada.	Qualitativo, metodologia da Teoria Fundamentada nos Dados
Aytona *et al.*/2022/Filipinas	Estimar a necessidade de força de trabalho em saúde com base em cargas de trabalho reais dos profissionais de saúde.	Misto, transversal
Van Heerden e Jenkins/2022/África do Sul	Explorar o papel dos agentes comunitários de saúde na prestação de cuidados paliativos em um subdistrito rural na África do Sul.	Qualitativo, descritivo-exploratório
Muench *et al.*/2022/Estados Unidos	Examinar a prescrição rural/urbana de medicamentos psiquiátricos para idosos com doença mental grave por provedores de cuidados primários e especialistas em saúde comportamental.	Quantitativo, descritivo retrospectivo
Martin-Misener/2020/Canadá	Examinar o contexto da prática e as responsabilidades dos enfermeiros que fornecem cuidados primários essenciais no Canadá rural e remoto.	Quantitativo, transversal
Yonge *et al.*/2019/Canadá	Explorar como os enfermeiros rurais e discentes definem e lidam com os riscos e os encargos das viagens rodoviárias em áreas rurais.	Qualitativo, colaborativo
Zakumumpa/2020/Uganda	Descrever como o papel dos enfermeiros se expandiu em instalações de saúde da Uganda durante a era de expansão da terapia antirretroviral.	Misto, transversal e casos múltiplos
Kobe *et al.*/2022/Estados Unidos	Examinar a implementação do *Advanced Comprehensive Diabetes Care*, uma intervenção de telessaúde abrangente e baseada em evidências para diabetes tipo 2.	Misto, estudo de implementação de intervenção
Garg *et al.*/2022/Índia	Avaliar a competência dos enfermeiros no estado indiano de Chhattisgarh.	Quantitativo
Burrows, Calleja e Cooke/2019/Austrália	Determinar as necessidades de suporte dos enfermeiros que prestam atendimento de emergência em ambientes rurais.	Qualitativo, revisão de escopo
Breton *et al.*/2023/Canadá	Identificar os perfis de acesso avançado dos provedores de Atenção Primária à Saúde.	Quantitativo, transversal
Neville *et al.*/2020/Austrália	Explorar como os idosos se expressam acerca do acesso aos serviços de saúde da comunidade rural.	Qualitativo, gerontologia narrativa
Nelson-Brantley *et al.*/2021/Estados Unidos	Descrever estratégias comuns e barreiras específicas da prática, adaptações e determinantes da implementação do rastreamento do câncer na Atenção Primária à Saúde rural após a adesão a uma estratégia de organização de cuidados de saúde responsável.	Qualitativo, estudo de casos múltiplos
Haykin *et al.*/2020/Gana	Explorar as percepções dos profissionais de saúde não médicos em relação à sua capacidade de gerenciar doenças cardiovasculares e as barreiras à implementação do pacote de intervenção HEARTS da Organização Mundial da Saúde nas instalações da iniciativa *Community-based Health Planning and Services.*	Qualitativo
Norman *et al.*/2023/Nova Zelândia	Explorar a experiência dos enfermeiros rurais diante das barreiras na prestação de cuidados de saúde para controle de peso.	Qualitativo, estudo de caso
Strachan *et al.*/2022/Escócia	Avaliar a implementação da função de enfermeiro avançado na Atenção Primária à Saúde e estabelecer o que funciona, para quem, por quê e em que contexto.	Qualitativo, estudo de casos múltiplos
Pace *et al.*/2018/Ruanda	Avaliar o impacto de treinamento acerca da detecção de câncer de mama sobre o conhecimento e habilidades de enfermeiros na Atenção Primária à Saúde e agentes comunitários de saúde rurais.	Quantitativo, avaliativo
Lee *et al.*/2016/Estados Unidos	Avaliar um programa de consulta eletrônica centralizada (*E-consult*) na acessibilidade de pacientes rurais e o impacto na avaliação e no tratamento da osteoporose.	Quantitativo, avaliativo
Osborn *et al.*/2022/Austrália	Avaliar a acessibilidade, a disponibilidade e a utilização de serviços de saúde comunitários para povos aborígenes e descrever fatores que contribuem para fornecer serviços de saúde eficazes na perspectiva do provedor.	Misto, estudo de caso
Coleman-Minahan *et al.*/2020/Estados Unidos	Examinar o interesse de clínicos de prática avançada em treinamento para fornecer medicamentos e aborto por aspiração no Colorado.	Quantitativo
Kwak, Hwang e Lee/2021/Coreia	Explorar os efeitos de um programa de gerenciamento de doenças crônicas utilizando o modelo de telemedicina médico-enfermeiro na Atenção Primária à Saúde e os efeitos na adesão à medicação e na qualidade de vida relacionada à saúde em pacientes com doenças crônicas de áreas rurais remotas.	Quantitativo, modelo quase experimental de prée pós-teste com grupo controle não equivalente
Amin *et al.*/2020/Índia	Identificar as necessidades para garantir a disponibilidade e a eficácia dos enfermeiros de Atenção Primária à Saúde na prestação de cuidados de saúde em áreas rurais.	Misto, abordagem do caminho rural
Johns, McAuliffe e Dorsett/2019/Austrália	Investigar a prestação de cuidados psicossociais para usuários em cuidados paliativos em comunidades rurais australianas, além de identificar barreiras e facilitadores de encaminhamentos para serviço social.	Qualitativo
Xue *et al.*/2017/Estados Unidos	Documentar as tendências temporais em modelos alternativos de cuidados primários a idosos e examinar o papel desses modelos no atendimento a idosos rurais com múltiplas condições crônicas.	Quantitativo, transversal
Foster at al./2018/Zambia	Avaliar os resultados de um programa de formação em práticas de liderança e gestão para enfermeiros rurais.	Misto, avaliativo
McCullough *et al.*/2020/Austrália	Descrever e explicar, na perspectiva dos enfermeiros, as ações e interações envolvidas na prestação de Atenção Primária à Saúde em comunidades remotas.	Qualitativo
Diema *et al.*/2021/Gana	Descrever as práticas de visita domiciliar em um distrito rural na região de Volta, em Gana.	Misto, transversal descritivo
Suk, Oh e Im/2018/Coreia do Sul	Examinar os níveis de competência cultural, empoderamento e empatia em enfermeiros visitantes que trabalham com famílias multiculturais rurais na Coreia do Sul, além de verificar os fatores que afetam a competência cultural.	Quantitativo, descritivo transversal
Utz *et al.*/2020/Marrocos	Avaliar a aceitabilidade da abordagem de triagem e gerenciamento de diabetes mellitus gestacional pelos provedores de cuidados de saúde.	Qualitativo, avaliativo
Hjertstrøm, Obstfelder e Norbye/2018/Noruega	Analisar os esforços dos líderes de enfermagem noruegueses no desenvolvimento de um serviço de emergência em saúde municipal rural.	Qualitativo, abordagem etnográfica
Lovero *et al.*/2019/África do Sul	Avaliar o progresso e os desafios na implementação da Política Nacional de Saúde Mental e Plano Estratégico da África do Sul 2013-2020.	Misto, avaliativo
Hungerford *et al.*/2016/Austrália	Explicitar os principais componentes do modelo de prática de enfermagem desenvolvido para atender às necessidades de saúde de uma comunidade de área remota e turística (sem médico clínico geral residente).	Qualitativo, estudo de caso
Singh *et al.*/2019/Índia	Avaliar as perspectivas dos provedores rurais sobre o gerenciamento e encaminhamento de gestantes com alto risco obstétrico ou com complicações.	Quantitativo, transversal
Jin *et al.*/2019/China	Explorar a associação entre os papéis expandidos e a satisfação no trabalho dos provedores de Atenção Primária à Saúde rural na China.	Misto, transversal
Roots e MacDonald/2014/Canadá	Identificar os resultados associados à função de enfermeiro na prática colaborativa na Atenção Primária à Saúde.	Qualitativo. estudos de casos múltiplos
Reid, Ervin e Kelly/2019/Austrália	Descrever o primeiro ano de implementação de um programa de médicos no ensino médio em um ambiente rural em Victoria, Austrália.	Qualitativo, descritivo com abordagem pragmática
Iliffe *et al.*/2014/Reino Unido	Adaptar e testar um modelo norte-americano de gestão de casos com base em cuidados primários para pessoas com demência.	Misto, estudo de casos múltiplos
Mothiba e Tladi/2016/África do Sul	Determinar os desafios enfrentados por enfermeiros ao implementar o Programa Expandido de Imunização em clínicas rurais no Distrito de Capricórnio, Província de Limpopo, África do Sul.	Qualitativo, descritivo exploratório
Pesut *et al.*/2017/Canadá	Testar um serviço de cuidados paliativos rural liderado por enfermeiros.	Misto, observacional de centro único
Biordi *et al.*/2015/Estados Unidos	Apresentar um modelo de oferta de serviços de saúde bucal em dois locais do Programa Especial de Nutrição Suplementar para Mulheres, Bebês e Crianças.	Quantitativo

Destacam-se os estudos de avaliação de intervenções cujos públicos-alvo foram pessoas com múltiplas condições crônicas, diabetes mellitus tipo 2 refratário, mulheres em rastreamento do câncer de mama e outras doenças mamárias, doenças cardiovasculares, demência, idosos com doença crônica avançada e crianças menores de cinco anos. Investigações que não contemplavam avaliação de intervenções abrangeram, em sua maioria, cuidados de pessoas com doenças crônicas ou idosos. Pesquisas de implementação de políticas e de programas também foram encontradas.

No Quadro 2, foram apresentadas as práticas em saúde e estratégias desenvolvidas pelos enfermeiros da APS rural, identificadas e categorizadas por meio da análise temática, seguidas pelo total de estudos que as comtemplaram.

**Quadro 2:** Práticas em saúde e estratégias desenvolvidas pelos enfermeiros que atuam na Atenção Primária à Saúde em territórios rurais, Vitória da Conquista, Bahia, Brasil, 2025

PRÁTICAS/ESTRATÉGIAS	TOTAL DE ESTUDOS
Coordenação do cuidado/gestão de casos	17
Práticas de promoção da saúde, prevenção de doenças e intersetoriais	15
Cuidado de pessoas com doenças crônicas	14
Práticas mediadas por Tecnologias de Informação e Comunicação	12
Visitas domiciliares	12
Atenção à saúde das mulheres	12
Prescrição medicamentosa e tratamento	11
Gerência de serviços	11
Práticas colaborativas com outros profissionais	8
Transferência de práticas entre profissionais	6
Atendimento das urgências/emergências	5
Cuidados paliativos	4
Assistência às pessoas que vivem com HIV	3
Preceptoria	1

A despeito da ampla atuação dos enfermeiros em áreas rurais, os estudos mostraram vários desafios para seu pleno desenvolvimento, conforme ilustrado na Figura 2.

**Figura 2:** Desafios enfrentados pelos enfermeiros na atuação na Atenção Primária à Saúde em territórios rurais, Vitória da Conquista, Bahia, Brasil, 2025

## DISCUSSÃO

De forma geral, por meio da análise temática dos estudos, é possível identificar um amplo escopo de práticas e estratégias empreendidas pelos enfermeiros. O contexto rural, aliado à falta ou rotatividade do profissional médico, interpõe ao enfermeiro da APS a necessidade do desenvolvimento de uma mosaico de práticas, muitas vezes sobrepostas, que exige conhecimentos e habilidades bastante diversos daqueles existentes em outros ambientes, o que justificaria a definição do “enfermeiro de área remota” como uma função especializada^(18)^.

A transferência de práticas (*task shifting* ou *skill mix*) compreende a assunção de funções de outros profissionais, decorrendo em ampliação do escopo e aumento do acesso aos serviços pelos usuários. Esse processo foi comum^(18-21)^ e considerado estratégico para qualificar o acesso à APS para populações rurais. Ocorria mesmo não formalizada no país, ou seja, práticas não contempladas no escopo legal dos enfermeiros, impelidos pelas necessidades específicas da ruralidade e ausência de outros profissionais^(19-21)^. Um exemplo foi a gestão dos cuidados de pessoas com HIV, incluindo prescrição da terapia antiretroviral (TAR) e procedimento cirúrgico de circuncisão, na Uganda, tradicionalmente atribuídas aos médicos^(19)^. Em experiências na Austrália, enfermeiros atuavam sozinhos em um serviço de urgência/emergência desenvolvendo ações de recuperação do paciente, triagem, estabilização e continuação do tratamento até a alta^(18)^. No Canadá, solicitavam e interpretavam exames, faziam diagnóstico, prescrições medicamentosas e encaminhamentos de forma independente^(21)^.

Comumente, enfermeiros desenvolviam funções não apenas de médicos, mas também de farmacêuticos, técnicos de laboratório, paramédicos, administradores, radiologistas, assistente sociais e fisioterapeutas, em condições de baixos salários e sobrecarga laboral^(21)^. Estudos sugerem revisar o escopo de prática dos enfermeiros quanto à transferência de tarefas, descrevendo suas funções e políticas, para formalizar essas práticas e garantir a segurança do paciente e qualidade do cuidado, mediante processos educativos adequados. Ademais, é recomendado aumento do número de enfermeiros para reduzir a sobreposição de funções^(19-21)^.

Iniciativas de práticas colaborativas dos enfermeiros com outros profissionais^(22-27)^ foram um achado e, quando interinstitucionais, operadas por videoconferências e reuniões regulares. Estratégias de articulação entre enfermeiros da APS e enfermeiros da assistência domiciliar, equipes de asilos e médicos clínicos gerais para o estabelecimento de um serviço de emergência rural com coordenação dos cuidados, em uma experiência norueguesa, facilitaram o acesso dos usuários e colaboraram para a integralidade da atenção^(25)^.

Práticas colaborativas entre enfermeiros e outros profissionais na APS envolveram: agentes comunitários de saúde (ACSs) e profissionais da equipe multiprofissional em visitas domiciliares, estudos de caso e grupos educativos no Brasil^(27)^; gerente de medicamentos em intervenções de telessaúde para pacientes com diabetes nos Estados Unidos^(22)^; médicos na prestação de serviços de saúde na escola na Austrália^(23)^; e diversos membros da equipe de APS em atividades de planejamento, avaliação, monitoramento de ações e cuidado compartilhado em outros cenários^(24-26)^. No caso canadense, práticas colaborativas incluíram cuidado compartilhado e resultaram em melhoria da comunicação interprofissional e do trabalho em equipe^(26)^.

Foi frequente o trabalho dos enfermeiros rurais em plantão noturno, sendo, muitas vezes, o único provedor disponível nesse período. Nos plantões, eram responsáveis por atendimentos de urgência, emergência, acompanhamento dos pacientes nos leitos de emergência até a estabilização e transporte de pacientes^(18,25,27,28)^.

Em inciativa norueguesa^(25)^, os enfermeiros, além de gerenciarem os atendimentos de emergência, operavam um centro de comunicação de emergência local, no qual era realizada a triagem dos pacientes, por telefone, para decidirem a necessidade de atendimento presencial ou aconselhamento, o que exigia um conjunto de habilidades e conhecimentos nem sempre contemplados por processos formativos. Apesar de raras iniciativas de educação continuada, merece destaque uma experiência da Escócia^(28)^ na qual entidades responsáveis pela gestão e assistência do Serviço Nacional de Saúde (*health boards*), em áreas remotas e rurais, ofertavam treinamento avançado de enfermagem específico para atendimentos de urgência/emergência.

O cuidado ofertado pelos enfermeiros a pessoas com doenças crônicas foi frequente^(18,19,22,26,29,30)^, assim como e idosos e cuidados paliativos. Os casos de assistência às pessoas que vivem com HIV ocorreram na Uganda^(19)^, na Zâmbia^(30)^ e no Canadá^(26)^, incluindo prescrição e aconselhamento da TAR, dispensação de medicamentos, triagem, coleta de sangue para exames laboratoriais, realização do teste de HIV, trabalho na sala de tratamento e gestão dos casos de tuberculose.

No caso ugandense, enfermeiros lideravam visitas domiciliares, nas quais era ofertada a TAR com o objetivo de aliviar clínicas de HIV e encargos de transporte dos pacientes^(19)^. Na experiência da Zâmbia, toda a equipe, incluindo ACSs e voluntários, foi treinada em aconselhamento e testes para infecções sexualmente transmissíveis, o que resultou em melhorias na acessibilidade aos serviços (e.g., aumento da oferta do teste de bebês expostos ao HIV de 60% para 83%)^(30)^.

Cuidados paliativos abrangiam ações como elaboração de plano de cuidados, cuidados psicossociais, visitas domiciliares, supervisão dos ACSs para monitoramento contínuo dos pacientes, atividade educativa com cuidadores e pacientes^(24,31)^. Na Noruega, enfermeiros da APS rural promoviam videoconferências regulares com especialistas para discussão da situação clínica de pessoas em cuidados paliativos, tomada de decisões conjuntas e esclarecimento de dúvidas, o que, por vezes, evitava deslocamento dos pacientes aos hospitais^(24)^.

Estudo piloto no Canadá^(31)^ testou um serviço paliativo rural para idosos liderado por enfermeiros. A enfermeira navegadora (*nurse-led navigation*) incorporou ao cuidado, além dos serviços de saúde, ações de assistência social (mobilização de recursos para transporte, refeições e limpeza) e articulação com redes de apoio. Foram ofertados aos idosos e cuidadores uma linha telefônica de plantão, disponível 24 horas, todos os dias da semana, para assistência de enfermagem entre as visitas, e um diário para registro de questões relevantes da saúde dos usuários, a fim de compartilhar com os outros provedores de cuidados. A enfermeira navegadora enviava relatórios aos médicos responsáveis com a evolução clínica e realizava encaminhamentos, quando necessário^(31)^.

Outra intervenção criada e liderada por enfermeiros para pessoas com múltiplas condições crônicas, nos Estados Unidos^(32)^, foi o *mI SMART*, uma estrutura de sensores e dispositivos móveis baseada na *web* por meio do *Chronic Care Model*. A funcionalidade do paciente do *mI SMART* combinava educação síncrona e assíncrona, lembretes para realizar automonitoramento, registro do automonitoramento com respostas automatizadas e pessoais de médicos, notificações para uso de medicamentos, portal de mensagens assíncronas, videoconferência para consultas de rotina, acesso a resultados laboratoriais, e *links* para realização de pesquisas. Cada usuário recebeu dispositivos para automonitoramento (balança, glicosímetro, monitor de pressão arterial e *tablet*) e pacote de dados da internet. Como resultado, os usuários rurais apresentaram melhoras nos níveis de glicose, pressão arterial e peso em um curto período de 12 semanas^(32)^.

Por meio de tecnologias de informação e comunicação, os enfermeiros recebiam orientações de um médico à distância para oferecer assistência a pacientes com doenças crônicas, realizar referência^(18)^, promover o automonitoramento e gerenciar medicamentos para pacientes com diabetes mellitus^(22)^, realizar consultas eletrônicas (*e-consult*) a idosos com fraturas^(33)^ e gerenciar casos de pessoas com demência, estabelecendo contato por telefone ou carta/e-mail^(34)^. Tais iniciativas melhoraram o engajamento na autogestão e a comunicação paciente/enfermeiro, promovendo maior conscientização sobre controle da doença crônica e aumento do acesso a tratamento, com diminuição da necessidade de deslocamentos.

A prática de visitas domiciliares por enfermeiros no contexto rural tinha como finalidade avaliar as condições socioeconômicas, identificar riscos, planejar os cuidados de acordo com a realidade e os recursos dos pacientes, educar sobre saúde, verificar o cumprimento do calendário vacinal, realizar vacinações, controlar o crescimento e o desenvolvimento das crianças e realizar ações curativas^(26,27,29,31,35-37)^. Em Gana, os enfermeiros realizavam visitas nos períodos da manhã, tarde e noite^(36)^, enfatizando o processo de prevenção de doenças. Na Coreia do Sul, enfermeiros visitantes foram os principais responsáveis por fornecer cuidados a famílias multiculturais rurais^(35)^.

Práticas dos enfermeiros relacionadas à atenção à saúde das mulheres foram um achado recorrente e que abrangeram consultas de pré-natal (incluindo diagnósticos e tratamentos), puerpério, planejamento familiar, partos assistidos, realização de aborto, e rastreamento e gestão do câncer do colo uterino (com realização de exame citopatológico itinerante) e de mama^(26,27,30,36,38-43)^.

No Marrocos, um programa de triagem e tratamento descentralizado de diabetes gestacional (DMG) foi implementado nas unidades primárias, nas quais os enfermeiros desenvolviam diversas funções, como consultas de pré-natal, rastreio e gestão da DMG, aconselhamento nutricional e contato via celular para esclarecimento de dúvidas e comunicação das marcações de consultas com especialistas. Com isso, reduziu-se o atraso na realização dos testes de triagem e aumentou-se a adesão ao programa e a satisfação das gestantes^(39)^.

Também foi identificada realização de aborto medicamentoso, com recomendação de ampliação dessa prática em áreas rurais, nas quais não há médicos clínicos especializados e que a unidade de APS pode ser de difícil acesso^(43)^.

Práticas de prescrições e tratamento foram frequentes^(19,21,25,36,40,41,44)^. Estudo nos Estados Unidos identificou que EPAs forneceram mais prescrições de medicamentos psiquiátricos do que médicos especialistas para usuários de áreas rurais^(44)^. A prescrição medicamentosa também foi presente na Uganda, na Índia, na Coreia, em Gana, na Noruega, na África do Sul e na Austrália, sendo destinada a tratamentos de febre, de diarreia, tuberculose, de doenças crônicas, de dores menores, de casos de urgência, TAR e tratamento de complicações precoces de gestantes^(19,21,25,36,40,41)^. Na Índia, os enfermeiros prescreviam medicamentos para diversas doenças e condições, como diarreia com desidratação grave, pneumonia, malária, hipertensão, diabetes, candidíase, pré-eclâmpsia, escabiose, intoxicação e anemia falciforme, como forma de melhorar o acesso da população^(41)^.

Os achados desta revisão evidenciam o protagonismo dos enfermeiros da APS de territórios rurais em papeis de liderança e gerência de serviços da APS, de clínicas de HIV e de unidades móveis (odontológicas, de triagem de câncer de mama e de osteoporose). Entre as atividades desenvolvidas no âmbito da gestão dessas unidades, destacam-se a gerência de recursos humanos, materiais e medicamentos, a oferta de treinamentos, a supervisão do setor de imunização, a promoção de canais de comunicação para usuários, a articulação com outros serviços de saúde para garantir cuidados integrados, a avaliação, o planejamento e a implementação de estratégias com o intuito de aprimorar a qualidade do atendimento prestado à comunidade^(19,20,22,23,25-27,30,44,45)^. Todavia, uma experiência brasileira demonstrou que a centralização da gerência das unidades de APS no enfermeiro era geradora de sobrecarga laboral^(45)^.

Promoção da saúde e prevenção de doenças estiveram presentes e abrangeram práticas de atividades educativas coletivas, estratégias para mobilizar a participação da comunidade no serviço, identificação de riscos no território, ações de imunização e execução de programas de promoção da saúde (saúde bucal, nutrição e exercícios)^(20,23,26,27,29-31,36)^. Iniciativas dos enfermeiros para ações intersetoriais englobaram ofertas de serviços em escolas e articulação da assistência social para a promoção do cuidado integral^(23,31,46)^.

Além das práticas e estratégias empreendidas por enfermeiros que atuam em áreas rurais, os estudos sinalizaram um conjunto de desafios que, por vezes, limitam o escopo de atuação. As especificidades da ruralidade exigem diversas habilidades e conhecimentos que raramente são ofertados aos profissionais por meio de processos formativos, seja na graduação, na educação continuada ou em treinamentos no serviço^(18,19,27-29,35,40,46)^. Esta foi uma questão-chave apontada nos estudos: a necessidade de formação adequada para os profissionais^(18-21,27,29,35,36,39-44,46-49)^. Apesar de escassas iniciativas, vale ressaltar o caso da Austrália, onde programas universitários e de educação continuada ofereciam treinamento e suporte para profissionais de saúde nas áreas rurais^(48)^. Na Índia, enfermeiros recebiam mentoria e suporte via teleconsulta^(50)^. De toda forma, profissionais relataram dificuldades de participação devido à sobrecarga laboral, à ausência de substitutos e a dificuldades de deslocamento.

A ausência de transporte para executar o trabalho na área rural^(27,29,36)^, intensificado por extensos deslocamentos em condições desfavoráveis (estradas danificadas e não sinalizadas, neve ou calor extremo)^(21,27-29,36,42)^ e pouca quantidade de enfermeiros para grandes populações geograficamente dispersas^(18-21,27,36,38,47)^, foi um desafio recorrente. Em alguns casos, os enfermeiros se deslocavam ou transportavam pacientes com recursos próprios.

Precariedade da infraestrutura dos serviços de APS, insuficiência de medicamentos, materiais e equipamentos foram observados nos estudos e influenciavam a qualidade do cuidado ofertado^(18,19,21,27-29,36,46,47)^. A falta de estrutura nas áreas rurais para enfermeiros e suas famílias, como moradia, oportunidades de emprego para cônjuges, escolas qualificadas para filhos^(50)^ e salários adequados^(19,27)^, emergiram como desafios para atuação dos enfermeiros em contexto de ruralidade. Em uma experiência indiana, buscou-se enfrentar tais problemas por meio da oferta de habitação, salário comparável ao de hospitais da cidade e uma unidade bem equipada para todos os enfermeiros^(50)^.

O exercício das práticas avançadas de enfermagem era limitado pela legislação, pela aceitação de médicos e outros profissionais, pela compreensão limitada da comunidade, pela ausência de supervisão e avaliação de competências e por escassas iniciativas de educação permanente, conforme já mencionado^(28,44)^. Em um caso específico nos Estados Unidos, os regulamentos estaduais para o exercício do EPA exigiam supervisão médica para a prática de prescrição medicamentosa^(44)^.

Os estudos sinalizaram relato recorrente acerca da sobrecarga de trabalho dos enfermeiros da APS em territórios rurais^(18,20,22,34,38,39,45-47)^. Esses profissionais desenvolviam funções que se acumulavam, a exemplo das assistenciais e gerenciais, e, em alguns momentos, eram o único provedor de cuidados primários, sem a valorização social e financeira compatível.

Problemas relacionados ao subfinanciamento, à indisponibilidade de serviços de saúde de suporte à APS, à ausência ou limitação de plano de carreira, às lacunas no sistema de informação, à deficiente coordenação e centralização da gestão distante do cotidiano das unidades de APS afetavam de forma negativa as possibilidades de atuação da enfermagem nas áreas rurais^(18,28,35,46)^.

### Limitações do estudo

A ausência do termo “escopo de práticas” ou “práticas de saúde” como DeCS/MeSH é considerada uma limitação do estudo, havendo necessidade do uso de descritores similares. A existência de diversos sistemas de saúde dificultou a delimitação dos serviços de APS nos diferentes países, assim como a definição da profissão de enfermagem, aqui considerada como formação de nível superior, dependendo do contexto. De toda forma, ressalta-se a importância do conjunto expressivo de evidências apresentado, considerando o reduzido número de estudos que abordam a temática da atuação do profissional de enfermagem em contexto rural no Brasil, apesar da extensão desses territórios, da distância dos centros urbanos, da rarefação populacional e das grandes desigualdades de acesso a bens e serviços públicos, entre os quais se encontra a saúde.

### Contribuições para a área da enfermagem, saúde ou políticas públicas

Os achados e conclusões deste estudo contribuem para o fortalecimento de políticas de saúde, especialmente da APS, bem como para a sustentação de modelos de práticas robustos e, por conseguinte, para a oferta de serviços mais equânimes, reconhecendo as especificidades dos territórios rurais. Ademais, pode colaborar para o aprimoramento da formação profissional dos enfermeiros e fomento de estratégias que qualifiquem e valorizem a sua atuação no contexto rural, além de contribuir para o movimento atual de debate acerca do escopo de práticas do enfermeiro.

## CONSIDERAÇÕES FINAIS

Enfermeiros em áreas rurais e remotas desempenham um papel essencial na ampliação do acesso à APS, no campo da assistência, promoção da saúde e prevenção de doenças, além da função de gestão de casos e gerência de unidades de saúde.

Embora o escopo de prática seja direcionado aos usuários da APS, os principais grupos beneficiados pelos cuidados dos profissionais de enfermagem que atuam em áreas rurais foram idosos, pessoas com doenças crônicas e em cuidados paliativos, segundo os estudos revisados, ou seja, grupos mais vulnerabilizados. As atividades incluíram consultas, diagnósticos, exames, prescrição, visitas domiciliares, gestão de casos, administração de unidades de saúde e ações intersetoriais para garantir a integralidade do cuidado.

A despeito do seu papel crucial para a viabilidade da APS em contexto rural, o desenvolvimento das práticas em saúde pelo enfermeiro enfrenta desafios que podem comprometer a qualidade do cuidado. À luz das potencialidades não apenas para ampliar, mas também apoiar o desenvolvimento das práticas de enfermagem, sobretudo nas áreas rurais, essenciais para garantir acesso à saúde a populações vulnerabilizadas, tornam-se fundamentais políticas públicas voltadas à melhoria da infraestrutura dos serviços de saúde, garantia de educação permanente, suporte profissional, e políticas de retenção e fixação, assegurando condições que favoreçam a atuação qualificada e resolutiva desses profissionais.

## Data Availability

Os dados de pesquisa estão disponíveis em repositório: https://data.mendeley.com/datasets/fyk4vsyrmx/1.
